# Gene pool sharing and genetic bottleneck effects in subpopulations of *Eschweilera ovata* (Cambess.) Mart. ex Miers (Lecythidaceae) in the Atlantic Forest of southern Bahia, Brazil

**DOI:** 10.1590/1678-4685-GMB-2018-0140

**Published:** 2019-11-14

**Authors:** Alesandro S. Santos, Daniela B. Borges, Caio V. Vivas, Cassio Van Den Berg, Polliana S. Rodrigues, Roberto Tarazi, Fernanda Amato Gaiotto

**Affiliations:** 1 Laboratório de Ecologia Aplicada à Conservação, Pós-Graduação em Ecologia e Conservação da Biodiversidade, Universidade Estadual de Santa Cruz, Ilhéus, BA, Brazil.; 2 Pós-Graduação em Genética e Biologia Molecular, Universidade Estadual de Santa Cruz, Ilhéus, BA, Brazil.; 3 Universidade Estadual de Feira de Santana, Departamento de Ciências Biológicas, Feira de Santana, BA, Brazil.; 4 Laboratório de Marcadores Moleculares, Centro de Biotecnologia e Genética, Universidade Estadual de Santa Cruz, Ilhéus, BA, Brazil.; 5 BASF Discovery Breeder, Trindade, GO, Brazil.

**Keywords:** Functional connectivity, tree species, chloroplast, founder effect, simple sequence repeat development

## Abstract

Forest loss and fragmentation are the main threats to the maintenance of the Atlantic Forest, an important global biodiversity hotspot. Because of the current critical level of deforestation, ecological corridors are needed to facilitate species dispersion and gene flow among fragments. This study was conducted to investigate the genetic variability and gene pool sharing of *Eschweilera ovata* in five forest remnants in southern Bahia, Brazil using nuclear simple sequence repeat (nSSR) and plastid simple sequence repeat (cpSSR) microsatellite markers. cpSSR marker analysis revealed the domains of four haplotypes, showing that 80% of the individuals had only four maternal origins, reflecting a founder effect and/or genetic bottleneck. The results of cpSSR and nSSR analyses indicated moderate genetic diversity, particularly in conservation units with full protection, which showed the best parameters of all areas evaluated. Another indication of the susceptibility of these populations to forest loss and fragmentation was the strong genetic bottleneck observed. In contrast, genetic structure analyses (F_ST_ and discriminant analysis of principal components) revealed gene pool sharing between the subpopulations, which may reflect the historical gene flow that occurred before forest fragmentation.

## Introduction

The Atlantic Forest in the east of Brazil is an important center of endemism associated with high species richness of several taxa, particularly tree species (Thomas *et al.*, 1998; Myers *et al.*, 2000; Martini *et al.*, 2007; Murray-Smith *et al.*, 2008). Despite its great importance for biodiversity conservation, this phytogeographical domain has been reduced to approximately 11–16% of its original area (Ribeiro *et al.*, 2009). This anthropogenic forest reduction represents one of the main threats to the permanence of species in this environment by reducing resources and increasing the risks of population extinctions (Pessoa *et al.*, 2016; Benchimol *et al.*, 2017). Additionally, the negative effects of forest reduction on biodiversity can be potentialized through interactions that cause structural isolation and decrease species dispersal and gene flow among forest remnants (Tabarelli *et al.*, 2004; Pardini *et al.*, 2010; Lima and Mariano-Neto, 2014).

Considering the conservation scope and current state of the reduction and fragmentation of the Atlantic Forest, it is important to establish ecological corridors between forest remnants (MMA, 2008) to allow the dispersion of species (Damschen *et al.*, 2006). Enabling gene flow between fragments and corridors decreases the deleterious effects caused by isolation and the reduction of forest area that decrease populations and their genetic diversity, thus increasing the risk of local extinctions over time (Carvalho *et al.*, 2015).

In the last few decades, genetic studies using a conservationist approach have mainly employed molecular markers such as microsatellites to evaluate the variability and distribution of the genetic diversity of tree species (Gaiotto *et al.*, 2003; Santos *et al.*, 2016; Carvalho *et al.*, 2017; Torres-Florez *et al.*, 2017). Some of these studies used microsatellite markers with biparental and uniparental inheritance patterns to determine historical colonization patterns and functional connections among tree species populations (Martins *et al.*, 2011; Zhang *et al.*, 2012). Several studies revealed founder effects in plant populations by using chloroplastidial microsatellites (cpSSRs), which show maternal inheritance in most species of angiosperms and act as functionally haploid and non-recombinant markers (Corriveau and Coleman, 1988; Weising and Gardner, 1999; Parducci *et al.*, 2001; Li *et al.*, 2012; Tong *et al.*, 2013). Additionally, studies combining nuclear and chloroplast microsatellite markers have shown similar results, suggesting that these approaches are complementary (Zhang *et al.*, 2012; Tóth *et al.*, 2017).

Because of the current critical state of the reduction and fragmentation of the Atlantic Forest, we evaluated the genetic variability and gene pool sharing of a tree species, *Eschweilera ovata* (Cambess.) Mart. ex Miers (Lecythidaceae), in five forest remnants in southern Bahia, Brazil, using cpSSR and nSSR markers. These remnants are in a region with high endemism and plant richness where efforts are underway to implement ecological corridors that connect forest fragments and conservation units (Fonseca *et al.*, 2004)*.* This tree species has a large occurrence (Atlantic Forest, Amazon Forest, Cerrado and Caatinga) and shows a zoochoric syndrome of seed dispersal and pollen. Additionally, it was suggested that this species can be used to promote the recovery of degraded areas. *E. ovata* is also economically exploited to make the berimbau, a musical instrument used in capoeira orchestras, a cultural symbol of the state of Bahia, Brazil (Lorenzi, 1998). In this study, we tested the following hypotheses: (1) The subpopulations of *E. ovata* have high genetic diversity in both molecular markers, considering the wide occurrence and density of the species; and (2) The subpopulations still share a gene pool, reflecting functional connectivity prior to forest loss and fragmentation.

## Material and Methods

### Study area

**Figure 1:**
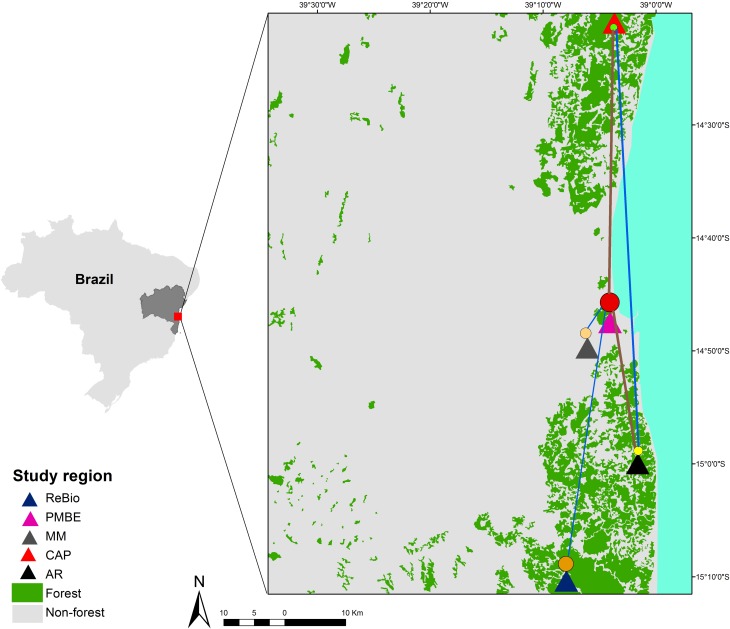
Map of Brazil. The state of Bahia is highlighted, indicating the region of the study, with the current forest cover. Blue and brown lines show network linkages identified by EDENetworks (Kivelä *et al.*, 2015) between nodes (sampling sites), using cpSSR markers. Line thickness is proportional to linkage strength and node size is proportional to the number of haplotypes for each sampling sites de *E. ovata*. Map data: Atlas of the Atlantic Forest remnants of the year 2016, obtained from SOS Mata Atlântica.

The study was conducted in five areas in southern Bahia, Brazil, a portion of the Atlantic Forest that is considered a priority for conservation actions due to its biological importance (Thomas *et al.*, 1998; Martini *et al.*, 2007). Using ArcGIS (10.2), we created a map of the study region that includes all areas and forest remnants (Figure 1). Four of the five locations are inside a protected area (PA): Reserva Biológica de Una (ReBio) covering 11,400 hectares in Una; Parque Municipal Boa Esperança (PMBE) covering 437 hectares in the urban area of Ilhéus; Reserva Particular do Patrimônio Natural (RPPN) Mãe da Mata covering 13 hectares in Ilhéus; and RPPN Capitão covering 660 hectares in Itacaré. The fifth area corresponds to an arboreal restinga (Restinga) between the PMBE and ReBio in Ilhéus, which is not in a PA.

In each study area, we collected leaf samples from 15 *E. ovata* adult individuals for genetic analysis and georeferenced the trees using GPS (GPS Garmin Map 62 s, Olathe, KS, USA). Sampling was performed to cover as much of each area as possible while avoiding the sampling of individuals geographically close to each other to reduce the chances of kinship between them and to sample the genetic pool of each collection area (distance varying between 10 and 2,000 m between individuals sampled). Estimates of the population genetics parameters are influenced by number and frequency of informative alleles (frequency ≥ 0.05) (Hale *et al.*, 2012). Thus, to evaluate the accuracy of estimated allelic frequencies for the 15 individuals sampled in each of the four subpopulations using nSSR markers, we calculated the allele frequencies and mean allelic pattern (number of different alleles, number different alleles with a frequency ≥ 5% and Shannon’s information index) with standard deviation using the GenAlex 6.5 program (Peakall and Smouse, 2012). If the number of individuals influenced the estimates mentioned above, great heterogeneity in these measures would be observed among the populations as a reflection of sampling error.

### DNA extraction, development of specific microsatellite primers, and genotyping with cpSSR and nSSR

DNA was obtained according to the CTAB protocol (Doyle and Doyle, 1987) and the quantification was performed in agarose gels (0.8%).

An enriched library was obtained by hybridization with (CT)_8_ and (GT)_8_ biotinylated probes, and positive fragments for the microsatellites were amplified and sequenced on an ABI 3100 genetic analyzer (Applied Biosystems, Foster City, CA, USA). We used Primer3 software (Untergasser *et al.*, 2007) to design primer pairs.

To characterize the new nuclear microsatellite marker set (nSSR) and perform genetic analysis of the populations, we used individual DNA sampled from four subpopulations (ReBio, MM, Cap, and AR) for a total of 60 trees. PCR samples contained 7.5 ng of genomic DNA, 10 μL of Top Taq Master Mix kit (Qiagen), 0.8 μM of primer reverse, 0.4 μM of forward primer, and 0.16 μM of M13 tail (CAC GACGTTGTAAAACGA) labeled with a fluorochrome (6-FAM, VIC, PET, or NED, Applied Biosystems, Foster City, CA, USA). The amplification reaction was run in a Veriti 96-Well Thermal Cycler (Applied Biosystems). It consisted of an initial denaturation step at 94 °C for 1 min followed by 30 cycles of an denaturation step at 94 °C for 1 min, annealing step at the specific annealing temperature for 45 s, and extension step at 72 °C for 1 min; 8 cycles for M13 tail amplification at 94 °C for 1 min, 53 °C for 45 s, and 72 °C for 1 min; followed by a final extension step at 72 °C for 10 min. The amplified fragments were subjected to capillary electrophoresis in a multiload system using an ABI 3500 genetic analysis (Applied Biosystems). Peaks were analyzed using GeneMarker software 2.6.2 (SoftGenetics, State College, PA, USA).

Ten loci were amplified in all sampled individuals (75 trees) from the five subpopulations (ReBio, MM, Cap, and AR) using chloroplastidial microsatellites (cpSSR) (Weising and Gardner, 1999) with an M13 tail and respective tail fluorophores (6FAM, VIC, NED, and PET, Applied Biosystems). Amplifications were performed by PCR in Life Pro, 96, gradient thermal cycler (TC-96/G/H (B) A) Bioer Technology, Tokyo, Japan). The final concentration of PCR reagents in 13 μL was: 0.75 ngμL^-1^ of genomic DNA; 0.325 mM of each dNTP (Invitrogen, Carlsbad, CA, USA); 1.3X PCR buffer (Invitrogen); 4.0 mM MgCl_2_; 0.325 mgμL^-1^ BSA (Invitrogen); 0.2 mM forward primer with the M13 tail; 0.4 mM reverse primer; 0.06 mM M13 complementary primer with the fluorophore; 1 U Taq DNA polymerase (Invitrogen), and MilliQ water to complete the final volume. PCR was performed under the following conditions: denaturation at 94 °C for 2 min followed by 30 cycles of denaturation at 94 °C for 1 min, annealing over a temperature gradient of 48 °C to 62 °C for each primer pair for 1 min, and extension at 72 °C for 1 min. The second PCR step was programmed for 8 cycles as follows: denaturation at 94 °C for 1 min, annealing at 53 °C (specified for the tail primers) for 1 min, extension at 72 °C for 1 min, and final extension at 72 °C for 7 min. Genotyping of the PCR products was performed on a multiload system (ABI 3130xl, Applied Biosystems), and GSLIZ500 was used as a size fragment standard (Applied Biosystems). To analyze the electropherograms and obtain the genotypes, GeneMarker version 2.2.0 software was used.

### Analysis of nuclear microsatellite markers (nSSR) and population genetics

To calculate the alleles per locus (A), probability of exclusion of paternity (Q), and identity index (I), we used the CERVUS 3.0.6 program (Kalinowski *et al.*, 2007). We used the divBasic function in the diveRsity package in the R software (Keenan *et al.*, 2013) to calculate allelic richness, expected heterozygosity (H_E_), observed heterozygosity (H_O_), and inbreeding coefficient (*f*). The confidence interval of H_E_, H_O_, and *f* at 95% was calculated using divBasic function with 10,000 bootstraps. To plot the results of these analysis, the ggplot2 package was used in R software (Wickham, 2010).

Analysis of molecular variance (AMOVA) was conducted using GenAlex 6.5 (Peakall and Smouse, 2012). To evaluate the genetic structure of the subpopulations analyzed, we calculated F_ST_ using the divBasic function of the diveRsity package (Keenan *et al.*, 2013) and discriminant analysis of principal components (DAPC) (Jombart *et al.*, 2010) using the Adegenet 2.0.0 package in R software (Jombart *et al.*, 2008). To calculate the 95% confidence interval of the F_ST_ with 10,000 bootstraps, we used the divBasic function in the diveRsity package in R software. Posteriorly, to determine if the geographic distance (km) influenced the genetic distance between populations (F_ST_), simple linear regression analysis was performed in R software (http://www.r-project.org/). To identify the number of clusters, we used the find.clusters function, retaining all main components, and the best fit cluster number was inferred through the Bayesian information criterion (BIC), selecting the lowest BIC as the ideal cluster. To describe the relationships between the identified clusters, the generic function DAPC was used, retaining the number of principal components that incorporated 80% of the cumulative variance (PCA eigenvalues) and number of discriminant functions (DA eigenvalues) to maximize variation between groups. In the graphical representation of the results obtained in DAPC analysis, we used the scatter function to assign individuals to clusters according to their gene pool and compoplot function to produce a diagram representing the genetic pool of the individuals associated with clusters of the subpopulations.

### Population genetic analysis with chloroplastid microsatellite markers (cpSSR)

To analyze the cpSSR loci, we used Haplotype Analysis software version 1.04 (Eliades and Eliades, 2009) to calculate the following genetic parameters: number of haplotypes (N_H_), number of unique haplotypes (P_H_), number of effective haplotypes (H_Ne_), haplotype richness (H), haplotypic diversity (H_E_), total genetic differentiation (F_ST_) between areas, and average genetic distance between individuals (D^2^sh). Linear regression analysis was performed in R software (http://www.r-project.org/) to detect associations between the pairwise genetic (F_ST_) and geographical distances (km).

To verify the existence of genetic bottlenecks in the subpopulations, we conducted the Wilcoxon test in software BOTTLENECK version 1.2.02 02 (Cornuet and Luikartt, 1996). As recommended for studies with few individuals per population and few loci (<20 loci), the Wilcoxon test with the infinite allele model (IAM), stepwise mutation model (SMM), and two-phase model (TPM), which allows multiple-step mutations, were applied (Piry *et al.*, 1999). For the TPM model, the proportion of SMM in TPM = 0.000 and variance of the geometric distribution for TPM = 0.36, corresponding to the most sensitive values for most microsatellites (Piry *et al.*, 1999).

To visualize and analyze the genetic relationships without assuming *a priori* a cluster of individuals or populations, we used the EDENetworks program (Kivelä *et al.*, 2015). Network analysis consisted of nodes (individuals or populations) linked according to their genetic relationships. While constructing networks of subpopulations and individuals, automatic thresholding was used. Automatic thresholding was detected by EDENetwork using the percolation threshold. The automatic thresholding used for network analysis was slightly below the percolation threshold so that the network remained connected. In the network at the subpopulation level, F_ST_ was used as distance with the automatic threshold (0.42). In the network at the individual level, allele sharing was used as a distance with automatic thresholding (0.14).

## Results

### Development of nSSR for *E. ovata* and population analysis

We developed 13 new nuclear primer pairs for *E. ovata* which had (i) an average of 5.6 alleles, (ii) H_O_ of 0.214–1.000, (iii) H_E_ of 0.198–0.878, and (iv) a fixation index (F) of -0.333–0.4763 (see Tables S1 and S2). The combined probability of exclusion (Q) of these 13 loci was 0.9 10^-9^ and identity index (I) was 3 28^-10^. The sequence, allele amplitude, and annealing temperature for each primer are shown in Table S1.

**Figure 2:**
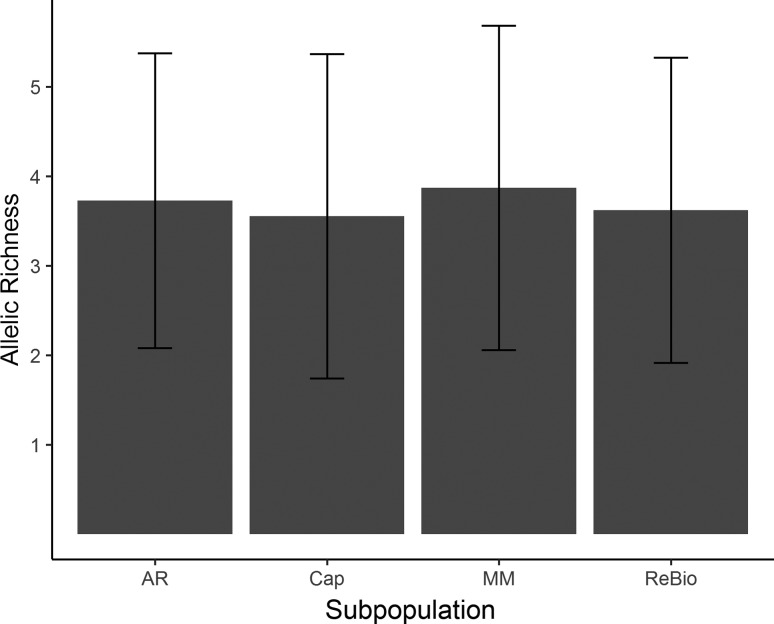
Allelic richness with standard deviation values for *E. ovata* subpopulations with nSSR markers.

**Figure 3:**
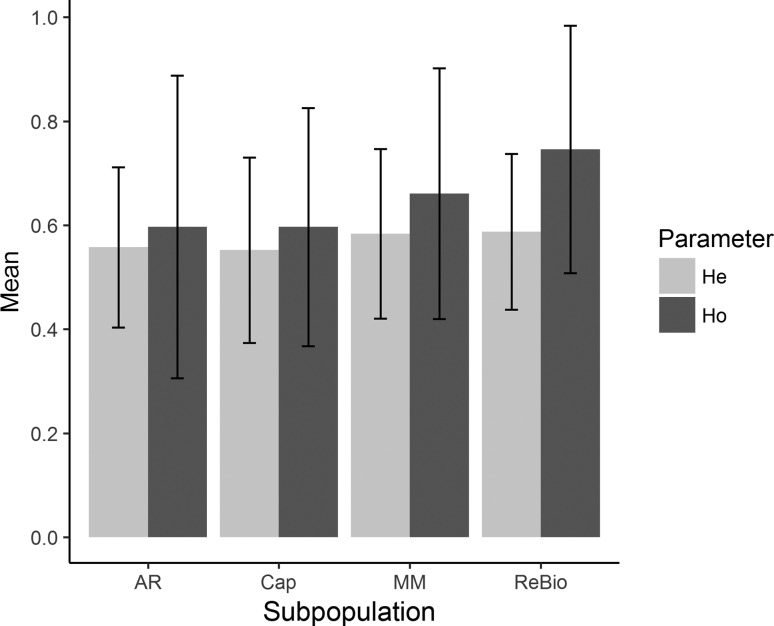
Observed heterozygosity (Ho) and expected heterozygosity (He) with standard deviation values for *E. ovata* subpopulations with nSSR markers.

**Figure 4:**
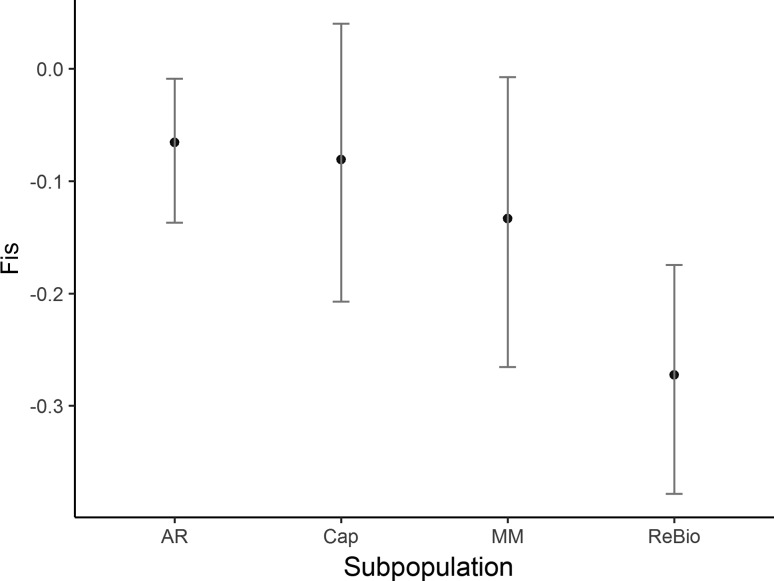
Fixation index (Fis) estimated values with nSSR markers and 95% confidence intervals based on 10.000 bootstrap values for *E. ovata* subpopulations with nSSR markers.

Population analysis verified that the 13 loci had similar distributions of allelic frequencies, average number of alleles, frequency of informative alleles, and estimated genetic diversities (Figures S1 and S2). The four subpopulations showed low numbers of effective alleles (AR = 2.610; MM = 2.883; Cap = 2.685; ReBio = 2.841), low allelic richness (Figure 2), and private alleles in all subpopulations (AR = 8, MM = 5, Cap = 4 and ReBio = 2). The observed heterozygosity (Ho) values were moderate to high (Figure 3). However, the Ho values were always higher than the expected heterozygosity (Figure 3), which was reflected by the negative values of the inbreeding coefficient (Figure 4) and significant genetic bottleneck in the four subpopulations (Table 1).

**Table 1:** Wilcoxon test for *E. ovata* subpopulations with nSSR markers.

Subpopulation	IAM	TPM	SMM
AR	0.04016*	0.55371	0.77258
Rebio	0.00012*	0.00201*	0.05493*
MM	0.00153*	0.07324	0.34241
Cap	0.00085*	0.07324	0.24866

**Figure 5:**
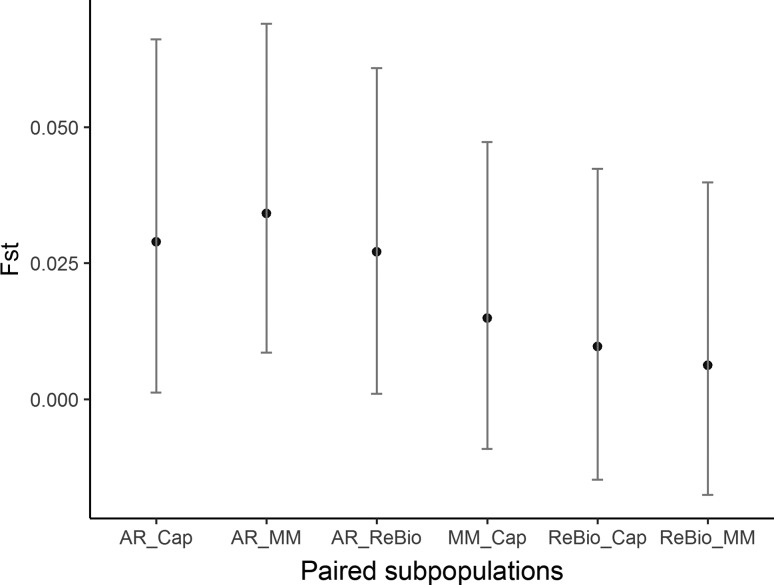
F_ST_ estimated values with nSSR markers and 95% confidence intervals based on 10.000 bootstrap values of *E. ovata* sampled in four subpopulations.

In contrast, in AMOVA, there were observed variances of 18% within and 80% among individuals, while there was only 2% variance among subpopulations. F_ST_ analysis revealed that subpopulations showed low and non-significant genetic differentiation (Figure 5), despite the relatively large distances between some subpopulations (up to 89 km).

**Figure 6:**
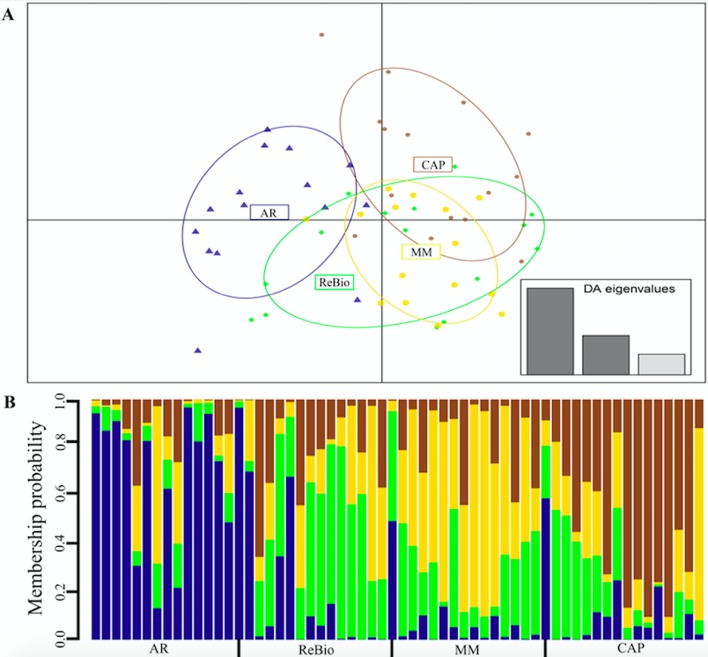
Genetic differentiation among the four *E. ovata* subpopulations based on nSSR markers. A) The graph represents the individuals as dots and the subpopulations as ellipses according to their original groups, which model 95% of the corresponding variability plotted. The subpopulations (ellipses) are plotted within the orthogonal space defined by the first two eigenvalues of the PCA (inserts). B) Diagram representing the gene pool of the individuals associated with each of the subpopulations. Each vertical bar represents an individual with the height of the column segments showing the probability of being assigned to one of the four subpopulations. Dark lines correspond to the collection location.

We verified that geographic distance did not explain the pattern of genetic distance between subpopulations, as these factors were not significantly correlated (R^2^ = 0.207, *p* = 0.36). DAPC revealed that for the four sampling areas form three genetic clusters (K = 3, BIC = 66.06), a group was formed by MM and ReBio and the other two groups formed separately in areas AR and CAP (Figure 6A), with sharing of the gene pool between groups (Figure 6B).

### Population analysis for *E. ovata* using cpSSR

Seven of the 10 cpSSR primers tested in *E. ovata* were successfully amplified. Three were monomorphic (cpSSR1, cpSSR3, and cpSSR7) and four were polymorphic (cpSSR2, cpSSR4, cpSSR5, and cpSSR6), with an average of three alleles per locus and 14 haplotypes. Of these haplotypes, four were found exclusively in PMBE (H1, H8, H9, and H14), two were exclusively found in ReBio (H3 and H4), one in AR (H5), and another was exclusively found in Cap (H11). In contrast, six of the 14 haplotypes (H2, H6, H7, H10, H12, and H13) were shared by two or more study areas. Dominance of the four haplotypes (H10, H6, H3, and H13) was observed in these areas, with 80% of the analyzed individuals presenting only four maternal origins (Table S3). In intra-population analysis, we detected an average of five haplotypes. The PMBE subpopulation contained the highest number (7) of haplotypes, while the RPPN Cap subpopulation had the lowest (3) (Table 2). When we analyzed the number of effective haplotypes, the five areas showed an average of 2.32, representing approximately half of the average number of haplotypes found. These subpopulations showed, on average, low richness (4) and haplotype diversity (0.55), with low genetic distances between individuals (0.70). Overall, the highest levels of genetic diversity were found in PMBE and ReBio (Table 2).

**Table 2:** E. ovata subpopulation genetic parameters based on the cpSSR markers.

Subpopulation	PA	AH	N	N_H_	P_H_	H_Ne_	H_R_	H_E_	D^2^sh
AR	Not	-	15	4	1	2.273	3	0.600	0.163
MM	Sustainable use	13	15	5	0	1.800	4	0.476	0.147
CAP	Sustainable use	660	15	3	1	1.316	2	0.257	0.041
ReBio	Integral protection	11,400	15	6	2	2.528	5	0.648	0.463
PMBE	Integral protection	437	15	7	4	3.689	6	0.781	2.707
**Mean**	-	-	15	5	1.6	2.321	4	0.552	0.704

**Figure 7:**
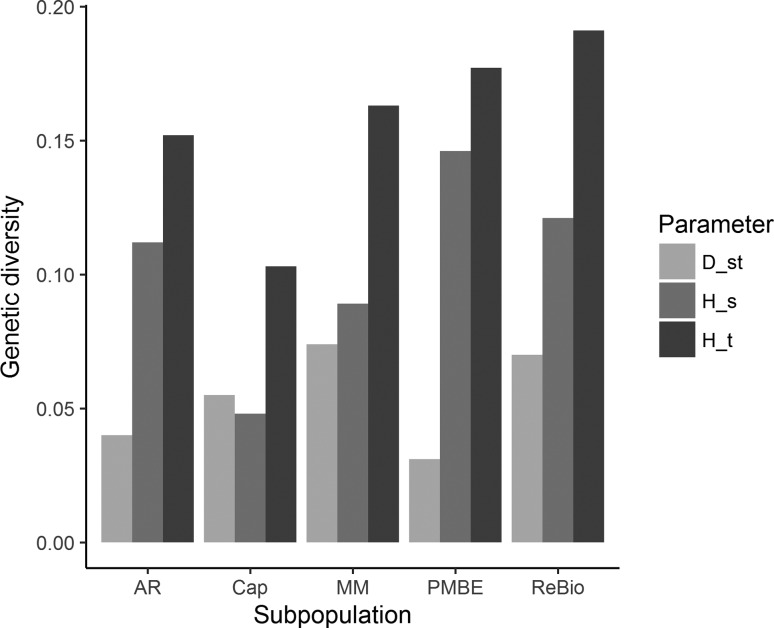
Genetic diversity partitioned from the *E. ovata* subpopulations with cpSSR markers. H_S_ = Genetic diversity within the population; D_ST_ = Diversity due to genetic differentiation; H_T_ = Total genetic diversity.

When we assessed the partitioned genetic diversity, we observed low levels of total genetic diversity (HT) and, consequently, low levels within subpopulations (HS), with higher values in PMBE and ReBio than in the other locations (Figure 7). Additionally, diversity due to genetic differentiation (D_ST_) was low in all subpopulations, with the lowest value in PMBE (Figure 7).

**Figure 8:**
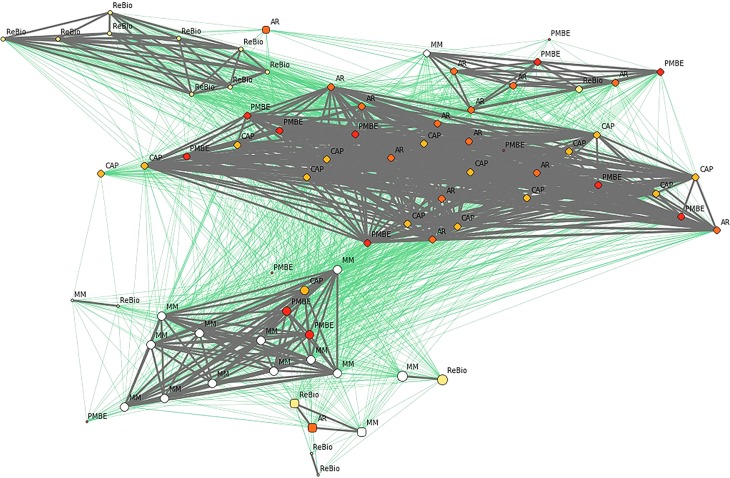
Network analysis among the individuals of *E. ovata* with cpSSR markers. Gray and green lines show network linkages identified by EDENetworks (Kivelä *et al.*, 2015) between nodes (individuals). The colors of the nodes represent the sampling sites, the line thickness is proportional to linkage strength and node size is proportional to the number of linkages for each node.

F_ST_ analysis revealed that subpopulations had low genetic differentiation (Table 3), despite the relatively large distances between some subpopulations (up to 89 km). Thus, geographic distance did not explain the pattern of genetic distance between subpopulations, as these factors were not significantly correlated (R^2^ = 0.08, *p* = 0.43). In network analysis at the population level, sharing of the gene pool was observed, reflecting the connection between subpopulations (Figure 1) and a substructure between individuals, forming two groups (gray and green) that shared alleles between individuals from different subpopulations (Figure 8).

**Table 3:** Genetic structure analysis (F_ST_) with cpSSR markers between areas in pairs in the lower diagonal and geographic distance in upper diagonal (km).

	AR	ReBio	MM	Cap	PMBE
AR	-	24	16	67	21
ReBio	0.046	-	37	89	43
MM	0.060	0.052	-	52	6
Cap	0.013	0.079	0.092	-	46
PMBE	0.005	0.035	0.036	0.015	-

## Discussion

We detected gene pool sharing among forest remnants by an insect-pollinated, animal-dispersed widely distributed tree in the Atlantic Forest. This information is important for recovering degraded areas, mainly in PAs. We also determined the relevance of integrating such important remnants in Atlantic Forest management plans, as suggested by ecological corridors projects in the region. Considering the difficulties of managing protected areas in Brazil because of the lack of knowledge about biodiversity in PAs (Oliveira *et al.*, 2017), conservation genetics can help public managers make decisions to maximize the potential of PAs in maintaining diversity and genetic connections between these areas and with surrounding forest remnants (Torres-Florez *et al.*, 2017).

The new set of nSSR markers developed for *E. ovata* showed polymorphisms sufficient for individual identification and kinship analysis, providing an excellent molecular tool for population genetic studies. Additionally, we demonstrated that the 15 individuals sampled and genotyped with 13 loci represented the number of alleles and frequency of informative alleles and genetic diversity of each of the four subpopulations. This small number of individuals may have been sufficient for population genetic analysis because geographically distant individuals were sampled within each subpopulation. Another important factor is that loci with high heterozygosity and a similar distribution of allelic frequency may enable genetic studies with reduced sample sizes (Hale *et al.*, 2012). Thus, this set of tools will be useful for subpopulation studies by providing access to information on the genetic variability of *E. ovata*; additionally, these data can be used to develop alternative strategies for managing and conserving tree species in the Atlantic Rainforest.

Genetic diversity assessment using the cpSSR marker revealed that the five subpopulations sampled have few haplotypes, with a large proportion of individuals (80%) showing only four haplotypes. This finding suggests that these subpopulations of *E. ovata* in the ecological corridor of the Atlantic Forest in southern Bahia were mainly founded by four maternal lineages, reflecting a small variety of seed sources and strong founding effect, or a recent genetic bottleneck (Parducci *et al.*, 2001; Logossa *et al.*, 2011; Li *et al.*, 2012; Wang, 2013). In contrast, the presence of unique haplotypes in the different subpopulations reflects the importance of the areas for maintaining genetic variability, particularly the integral protection area (PMBE and ReBio) that contains six of the eight unique haplotypes.

Intrapopulational analysis showed that *E. ovata* subpopulations have low average haplotypic diversity compared to other plants species (Petit *et al.*, 2005). Additionally, these populations have a low number of effective haplotypes and low haplotypic richness, reflecting the low genetic distance among individuals (Wang *et al.*, 2011; Wójkiewicz and Wachowiak, 2016). These results may be a consequence of the narrow genetic base of individuals that founded the subpopulations or a genetic bottleneck, reinforcing the importance of maintaining functional connectivity through gene flow between these subpopulations, as they may be more sensitive to external disturbances and prone to genetic drift (Parducci *et al.*, 2001; Martins *et al.*, 2011; Tong *et al.*, 2013). Taking into account the critical state of fragmentation and reduction of the Atlantic Forest (Ribeiro *et al.*, 2009), one method of maintaining contemporary gene flow is by managing regional land use to produce a heterogeneous mosaic (e.g., through ecological corridors or step-stones between forest remnants) (Fonseca *et al.*, 2004; Damschen *et al.*, 2006; Brudvig *et al.*, 2009).

The four subpopulations evaluated with the nSSR loci showed genetic diversity (H_E_ and H_O_) estimates similar to those found in rare and overexploited palm and tree species from the Atlantic Forest in southern Bahia, such as *Euterpe edulis* Mart. (H_E_ = 0.64 and H_O_ = 0.58) and *Melanoxylon brauna* Schott (H_E_ = 0.57 and H_O_ = 0.53) (Borges *et al.*, 2014; Santos *et al.*, 2015). However, these diversity indices are low compared to those of other more abundant species in the same environment, such as *Licania hypoleuca* Benth. (H_E_ = 0.84 and H_O_ = 0.71) (França *et al.*, 2015). It is also important to emphasize that the H_O_ of *E. ovata* was larger than the H_E_, indicating an imbalance between evolutionary forces, such as inbreeding or genetic bottleneck. Notably, inbreeding (*f*) values were negative in all subpopulations, strongly indicating that preferential crosses occurred between unrelated individuals. We used the Wilcoxon test to evaluate whether this excess in H_O_ was also the result of a genetic bottleneck in *E. ovata* subpopulations. The genetic bottleneck may cause the development of an excess of transient heterozygosity after a recent change in the effective population size when heterozygotes have a selective advantage, resulting in a higher H_O_ than H_E_ (Cornuet and Luikartt, 1996). The four subpopulations evaluated showed excess heterozygosity because of a genetic bottleneck, as revealed by the IAM. As demonstrated by Cornuet and Luikartt, (1996), the IAM is a more sensitive model for detecting the genetic bottleneck than the SMM and TPM models, indicating that the nSSR loci of *E. ovata* satisfied the requirements of the infinite allele model.

The results obtained with the two sets of markers (nSSR and cpSSR) showed convergence, with the results of AMOVA (nSSRs) and haplotype diversity analysis (cpSSR) revealing that diversity within the subpopulations contributed more to the overall composition than genetic variation among the subpopulations. This is likely the consequence of allele sharing and ancestral haplotype lineages that founded these populations (Wang, 2013). Additionally, areas of integral protection may play an important role in maintaining genetic variability. Human interference is prohibited in these areas, which may have favored the maintenance of unique haplotypes and alleles (Bruner *et al.*, 2001). Another interesting point is that although the differences were small, the areas of integral protection (ReBio and PMBE), showed the largest genetic indices compared to the other areas. The lowest genetic indices observed in the two sustainable use PAs may reflect the selective logging of *E. ovata* in RPPN Cap and forest reduction in RPPN MM, which both had anthropogenic impacts before the implementation of PAs (personal communication from the land owners). Genetic variability plays an important role in natural selection by maintaining the ability of populations to adapt to their habitats over time (Leimu and Fischer, 2008). Even small losses of genetic variability require attention, particularly in the subpopulation of Restinga (AR) that is outside the conservation unit and, consequently, more susceptible to human impact and the loss of alleles and haplotypes.

Genetic structure analysis (F_ST_) revealed that the studied subpopulations exhibited very low genetic structuring when analyzed with both markers, and this genetic pattern was not influenced by geographic distance. However, DAPC analysis revealed that the four collection areas formed three genetic clusters. However, many individuals from an *a priori* subpopulation are inserted into ellipses of other subpopulations, and there are overlaps of ellipses between subpopulations, supporting the observed low F_ST_ values. Additionally, there is a large mixture of the gene pool in individuals of different clusters, reinforcing the results obtained in F_ST_ analysis and showing that genetic diversity is mainly contained among individuals, as indicated by AMOVA.

Network analysis revealed a substructure between individuals, with two groups (green and gray), formed by individuals from different subpopulations. Importantly, the connections between individuals from the two groups demonstrated that although the individuals belonged to a specific group (green or gray), they also had genetic similarities with individuals from the other group. Thus, although two groups were observed, they are genetically connected, which may reflect allele sharing among individuals in these groups. Overall, the same patterns in the results were observed by DAPC (nSSR), network analysis (cpSSR), and Fst analyses with both markers revealing that these subpopulations are functionally connected by the dispersion of pollen and seeds. However, even with gene pool sharing, it is possible that gene flow among these subpopulations is currently impaired because of fragmentation of the forest in this specific landscape (see details in Figure 1). However, additional studies evaluating contemporary gene flow are needed to evaluate this issue. Furthermore, large infrastructure projects of the Brazilian government, such as construction of the southern port and the west-east railroad, may negatively impact some of the study areas (mainly PMBE and Cap), causing suppression and forest fragmentation and showing great potential for disorderly urban expansion. It is important to consider in the context of conservation during regional planning that these remnants of the Atlantic Forest maintain functional (genetic) connectivity. To implement efficient conservation strategies, we recommend that the five studied subpopulations should be considered as a single ecological entity during regional planning of different land uses.

## Supplementary material

The following online material is available for this article

Table S1 Characterization of the new 13 microsatellite loci specific to *E. ovata*.

Table S2 Characterization of 13 microsatellite loci in four subpopulations of *E. ovata*.

Table S3 Frequency of *E. ovata* haplotypes in the five study areas.

Figure S1 Allele frequencies of the 13 nSSR loci in four subpopulations of *E. ovata*.

Figure S2 Mean allelic pattern with standard deviation of the 13 nSSR loci in four subpopulations of *E. ovata*.

*Associate Editor: Dario Grattapaglia*
