# Supplementary material for: Gene pool sharing and genetic bottleneck effects in subpopulations of Eschweilera ovata (Cambess.) Mart. ex Miers (Lecythidaceae) in the Atlantic Forest of southern Bahia, Brazil
Source: Genet Mol Biol. 2019 Nov 14;42(3):655–65. doi: 10.1590/1678-4685-GMB-2018-0140 (PMC6905441; doi:10.1590/1678-4685-GMB-2018-0140)
Supplement: Supplementary file 1 [file 1415-4757-GMB-42-3-2018-0140-suppl1.pdf]

Supplementary Material to "Gene pool sharing and genetic bottleneck effects in subpopulations of *Eschweilera ovata* (Cambess.) Mart. ex Miers (Lecythidaceae) in the Atlantic Forest of southern Bahia, Brazil"

Table S1 - Characterization of the new 13 microsatellite loci specific to *E. ovata*.

Locus	Primer sequence (5'-3')	T (ºC)	Allele size range	Na	HWE
**EO04**	F: TTAgAgTTggTgAgTgCATATT	58	120-190	12	ns
	R:CAgCAACCTCTAgCATACTgT				
**EO07**	F: TgTgCTTACgCgTggACTAC	48	130-190	5	ns
	R: AgACCgAgAgCTACAggATCA				
**EO11**	F: AgTTgTCTgAAACCATCACT	48	300-400	2	ns
	R: CAACACAgCCCTACAAA				
**EO16**	F: CgCTgCTATCAACgAgACTg	58	170-230	2	ns
	R: TgCTATCTgAggCTgACACg				
**EO24**	F: TAgACTCCggCATgATAC	58	160-230	3	ns
	R: AACTCAgTgAATAACAATACAgT				
**EO25**	F: gAACACTgCTggAgTgATTATT	58	110-160	4	ns
	R: gTgAgTgCATTCTTCTgCAA				
**EO26**	F: CTTggAACCTgACCAgCAgT	58	260-320	8	ns
	R: TCAACAgAACCgATTCACACA				
**EO29**	F: TTCTgACTCTggTgTTgATT	48	240-310	8	ns
	R: CACAATTAgAggCAgAATCC				
**EO31**	F: ATggATAATTCCTCCgATgg	58	170-270	4	ns
	R: ACgCATgCATgATgAgATAC				
**EO39**	F: CAgCgAggAATggACgATA	58	220-270	4	ns
	R: TAgAgCAgAgCCTgATCgTg				
**EO40**	F: AgCATgATACgTTCTgTTggAg	58	200-260	5	ns
	R: gCACgAgAACCgAgTCTgTA				
**EO47**	F: TTCTgTTAAgCTCTACATgTCTgATT	60	250-330	3	ns
	R: CATCTgTCTgTCggCTgAg				
**EO63**	F: TTCCTgAAATCTggTTTCgTTT	58	270-340	13	ns
	R: CgCCgTATCAgAgCCATAAT				

Note: T (ºC) = Annealing temperature; Na = Number of alleles; HWE= Hardy–Weinberg equilibrium; ns=Not significant.
