# Supplementary material for: Gene pool sharing and genetic bottleneck effects in subpopulations of Eschweilera ovata (Cambess.) Mart. ex Miers (Lecythidaceae) in the Atlantic Forest of southern Bahia, Brazil
Source: Genet Mol Biol. 2019 Nov 14;42(3):655–65. doi: 10.1590/1678-4685-GMB-2018-0140 (PMC6905441; doi:10.1590/1678-4685-GMB-2018-0140)
Supplement: Supplementary file 2 [file 1415-4757-GMB-42-3-2018-0140-suppl2.pdf]

Supplementary Material to "Gene pool sharing and genetic bottleneck effects in subpopulations of *Eschweilera ovata* (Cambess.) Mart. ex Miers (Lecythidaceae) in the Atlantic Forest of southern Bahia, Brazil"

Table S2 - Characterization of 13 microsatellite loci in four subpopulations of *E. ovata*.

	**AR**					
**Loci**	**A**	**H_O**	**H_E**	**Q**	**I**	**F**
EO04	7	0.286	0.841	0.808	0.062	0.4763
EO07	5	0.733	0.674	0.619	0.161	-0.0861
EO11	2	0.267	0.239	0.174	0.618	-0.0672
EO16	2	1	0.517	0.281	0.375	-0.333
EO24	5	0.467	0.503	0.482	0.287	-0.0187
EO25	4	0.6	0.469	0.362	0.355	-0.1633
EO26	5	0.533	0.584	0.45	0.264	0.0504
EO29	4	0.133	0.605	0.447	0.257	0.6343
EO31	2	0.467	0.434	0.255	0.425	-0.0526
EO39	3	1	0.646	0.492	0.212	-0.2682
EO40	4	1	0.683	0.545	0.182	-0.2211
EO47	3	0.8	0.522	0.334	0.343	-0.2383
EO63	10	0.467	0.811	0.812	0.066	0.2722
	**ReBio**					
**Loci**	**A**	**H_O**	**H_E**	**Q**	**I**	**F**
EO04	7	0.714	0.878	0.857	0.043	0.0846
EO07	5	0.933	0.68	0.636	0.152	-0.2352
EO11	2	0.533	0.405	0.245	0.447	-0.1534
EO16	2	1	0.517	0.281	0.375	-0.333
EO24	4	0.867	0.618	0.511	0.217	-0.2281
EO25	2	0.333	0.37	0.232	0.476	0.0354
EO26	4	0.667	0.637	0.563	0.191	-0.0614
EO29	5	0.733	0.749	0.674	0.121	-0.0321
EO31	3	0.667	0.48	0.32	0.368	-0.1914
EO39	2	0.933	0.515	0.281	0.376	-0.3042
EO40	4	1	0.756	0.655	0.122	-0.1605

	AR					
EO47	2	1	0.517	0.281	0.375	-0.333
EO63	8	0.333	0.775	0.734	0.097	0.404
	**MM**					
**Loci**	**A**	**H_O**	**H_E**	**Q**	**I**	**F**
EO04	9	0.6	0.874	0.878	0.037	0.1767
EO07	5	0.8	0.536	0.561	0.204	-0.2046
EO11	2	0.267	0.198	0.174	0.618	-0.0672
EO16	2	1	0.519	0.281	0.375	-0.333
EO24	4	0.667	0.628	0.552	0.197	-0.0264
EO25	3	0.467	0.362	0.385	0.302	0.0674
EO26	6	0.5	0.742	0.673	0.126	0.1564
EO29	5	0.733	0.655	0.536	0.2	-0.0831
EO31	4	0.5	0.423	0.396	0.34	-0.0385
EO39	2	1	0.537	0.281	0.375	-0.333
EO40	5	0.933	0.712	0.629	0.144	-0.1609
EO47	2	0.8	0.476	0.275	0.386	-0.2499
EO63	7	0.333	0.785	0.804	0.066	0.4069
	**Cap**					
**Loci**	**A**	**H_O**	**H_E**	**Q**	**I**	**F**
EO04	10	0.533	0.874	0.868	0.041	0.2281
EO07	3	0.600	0.536	0.403	0.299	-0.0967
EO11	2	0.214	0.198	0.151	0.672	-0.0507
EO16	2	1.000	0.519	0.281	0.375	-0.3330
EO24	3	0.667	0.628	0.482	0.223	-0.0449
EO25	3	0.286	0.362	0.275	0.464	0.0922
EO26	5	0.692	0.742	0.663	0.127	0.0053
EO29	6	0.533	0.655	0.582	0.183	0.0959
EO31	2	0.571	0.423	0.251	0.434	-0.1664
EO39	3	0.857	0.537	0.341	0.334	-0.2586
EO40	4	0.786	0.712	0.598	0.156	-0.0720
EO47	2	0.714	0.476	0.268	0.398	-0.2173
EO63	6	0.308	0.785	0.731	0.095	0.4158

Note: AR= Restinga; ReBio= Reserva Biológica de Una.; MM= RPPN Mãe da Mata; Cap= RPPN Capitão; A= Number of alleles, H_O= observed heterozygosity, H_E= expected heterozygosity, Q= probability of paternity exclusion, i= identity index; F= fixation index.
