# Supplementary material for: Gene pool sharing and genetic bottleneck effects in subpopulations of Eschweilera ovata (Cambess.) Mart. ex Miers (Lecythidaceae) in the Atlantic Forest of southern Bahia, Brazil
Source: Genet Mol Biol. 2019 Nov 14;42(3):655–65. doi: 10.1590/1678-4685-GMB-2018-0140 (PMC6905441; doi:10.1590/1678-4685-GMB-2018-0140)
Supplement: Supplementary file 3 [file 1415-4757-GMB-42-3-2018-0140-suppl4.pdf]

Supplementary Material to "Gene pool sharing and genetic bottleneck effects in subpopulations of *Eschweilera ovata* (Cambess.) Mart. ex Miers (Lecythidaceae) in the Atlantic Forest of southern Bahia, Brazil"

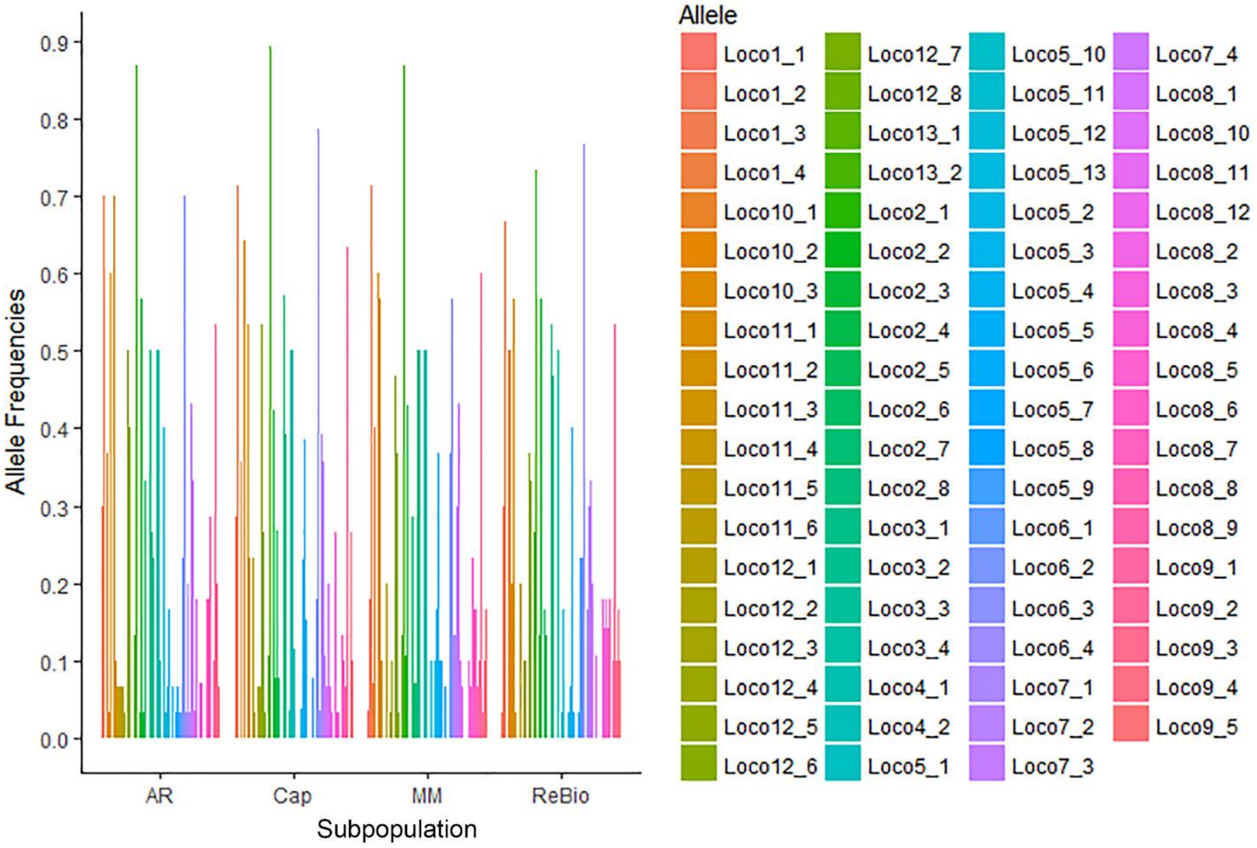

Figure S1 - Allele frequencies of the 13 nSSR loci in four subpopulations of *E. ovata*.
