# Supplementary material for: Gene pool sharing and genetic bottleneck effects in subpopulations of Eschweilera ovata (Cambess.) Mart. ex Miers (Lecythidaceae) in the Atlantic Forest of southern Bahia, Brazil
Source: Genet Mol Biol. 2019 Nov 14;42(3):655–65. doi: 10.1590/1678-4685-GMB-2018-0140 (PMC6905441; doi:10.1590/1678-4685-GMB-2018-0140)
Supplement: Supplementary file 5 [file 1415-4757-GMB-42-3-2018-0140-suppl3.pdf]

Supplementary Material to "Gene pool sharing and genetic bottleneck effects in subpopulations of *Eschweilera ovata* (Cambess.) Mart. ex Miers (Lecythidaceae) in the Atlantic Forest of southern Bahia, Brazil"

Table S3 - Frequency of *E. ovata* haplotypes in the five study areas.

Haplotypes	²Combination	NI	AR	ReBio	MM	PMBE	Cap
**H1**	222 159 130 124	1				0.067	
**H2**	227 159 125 124	2		0.067	0.067		
**H3**	227 159 126 124	9		0.600			
**H4**	227 160 125 124	2		0.133			
**H5**	227 160 126 124	1	0.067				
**H6**	228 159 125 124	14			0.733	0.133	0.067
**H7**	228 159 125 125	2		0.067	0.067		
**H8**	228 159 125 127	1				0.067	
**H9**	228 159 126 123	1				0.067	
**H10**	228 159 126 124	29	0.600			0.467	0.867
**H11**	228 159 126 125	1					0.067
**H12**	228 160 125 124	3	0.067	0.067	0.067		
**H13**	228 160 126 124	8	0.267	0.067	0.067	0.133	
**H14**	228 160 126 125	1				0.067	
**Total**		75	1.000	1.000	1.000	1.000	1.000

Note: AR= Restinga; ReBio= Reserva Biológica de Una; MM= RPPN Mãe da Mata; PMBE= Parque Municipal Boa Esperança; Cap= RPPN capitão; ²Combination = combination of alleles in the polymorphic cpSSR; NI= Number of individuals with the haplotype.
